# Idiopathic pulmonary fibrosis - a systematic review on methodology for the collection of epidemiological data

**DOI:** 10.1186/1471-2466-13-53

**Published:** 2013-08-20

**Authors:** Jaana Kaunisto, Eija-Riitta Salomaa, Ulla Hodgson, Riitta Kaarteenaho, Marjukka Myllärniemi

**Affiliations:** 1 Division of Medicine, Pulmonary Diseases, Turku University Hospital, Turku, Finland; 2 Department of Pulmonary Diseases and Clinical Allergology, University of Turku, Turku, Finland; 3 Division of Pulmonary Medicine, Heart and Lung Center, Helsinki University Central Hospital, Helsinki, Finland; 4 Center for Medicine and Clinical Research, Division of Respiratory Medicine, Kuopio University Hospital, Kuopio, Finland; 5 Unit of Medicine and Clinical Research, Pulmonary Division, University of Eastern Finland, Kuopio, Finland; 6 Respiratory Research Unit and Medical Research Center Oulu, Oulu University Hospital, Oulu, Finland; 7 Departement of Clinical Medicine, Division of Pulmonary Medicine, University of Helsinki, Biomedicum, PoBox 63, 00014, Helsinki C405b, Finland

**Keywords:** Interstitial lung disease, Incidence, Prevalence, Mortality, Register

## Abstract

**Background:**

Recent studies suggest that the incidence of idiopathic pulmonary fibrosis (IPF) is rising. Accurate epidemiological data on IPF, however, are sparse and the results of previous studies are contradictory. This study was undertaken to gain insight into the various methods used in the epidemiological research of IPF, and to get accurate and comparable data on these different methodologies.

**Methods:**

A systematic database search was performed in order to identify all epidemiological studies on IPF after the previous guidelines for diagnosis and treatment were published in 2000. Medline (via Pubmed), Science Sitation Index (via Web of Science) and Embase databases were searched for original epidemiological articles published in English in international peer-reviewed journals starting from 2001. After pre-screening and a full-text review, 13 articles were accepted for data abstraction.

**Results:**

Three different methodologies of epidemiological studies were most commonly used, namely: 1) national registry databases, 2) questionnaire-based studies, and 3) analysis of the health care system’s own registry databases. The overall prevalence and incidence of IPF varied in these studies between 0.5–27.9/100,000 and 0.22–8.8/100,000, respectively. According to four studies the mortality and incidence of IPF are rising.

**Conclusions:**

We conclude that there are numerous ways to execute epidemiological research in the field of IPF. This review offers the possibility to compare the different methodologies that have been used, and this information could form a basis for future studies investigating the prevalence and incidence of IPF.

## Background

Diagnostics and treatment of idiopathic pulmonary fibrosis (IPF) is currently undergoing changes. In the most recent guidelines [1], the diagnostic criteria of IPF are defined more accurately than they have been previously. Simultaneously, IPF treatment is undergoing the biggest shift in its history; the first drug intended for the prevention of IPF progression, pirfenidone, is entering clinical use in Europe, Canada and Asia [2,3]. In addition, an interim publication of the PANTHER trial revealed that the commonly used triple-drug treatment, namely prednisolone, azathioprine and N-acetylcysteine may be harmful to IPF patients, since compared to those who received placebo, individuals receiving triple-drug therapy had increased mortality [4].

Epidemiological data of IPF are sparse, and moreover, changes in classification of idiopathic interstitial pneumonias (IIP) and diagnostics make the comparison of the data from various decades difficult. Until the 1990s, idiopathic pulmonary fibrosis covered a heterogeneous group of different interstitial diseases. The ATS/ERS consensus statement of diagnosis and treatment in 2000 [5] and classification of IIPs in 2002 [6] defined IPF more accurately than it had been previously. In the new guideline radiological and histological criteria of UIP (usual interstitial pneumonia) are defined more precisely than before [1]: If high-resolution computed tomography (HRCT) features reveal a typical UIP pattern, IPF can be diagnosed without histological analysis, whereas if HRCT features show either possible UIP or no UIP features, a surgical lung biopsy should be taken. Major and minor criteria are no longer in use.

National IPF registries are being set up [7,8], and will most likely yield important data on IPF epidemiology after the diagnostic criteria revision. Therefore it is crucial to summarize the existing data from 2001–2011 for comparative purposes, and for a critical evaluation of epidemiological methods that can be put into practice in future studies.

## Methods

Our aim was to review the literature of IPF epidemiology that has been published after the previous international statement on IPF in 2000. Therefore, we performed a systematic literature search in September 2012 with the help of an information specialist at the University of Turku/Medical library. Relevant literature was identified through the databases Medline (via Pubmed) and Science Citation Index (via Web of Science) starting from 01/01/2001 onwards. Only articles published in English were included in the study. The search was limited to human adults, using the terms idiopathic pulmonary fibrosis, interstitial lung diseases and epidemiology, combined with geographical areas (Americas, Australia, Europe, Asia and cities). The initial search resulted in 214 articles. Two authors (JK, MM) independently pre-screened titles and abstracts, and data extraction resulted in 28 articles. All articles containing demographic data, incidence, prevalence or mortality rates of IPF or interstitial lung diseases (ILD), also including IPF patients, were accepted.

The 28 eligible articles were then full-text reviewed by four authors (JK, RK, E-RS and MM). By consensus, only international peer-reviewed original articles were included, leading to the exclusion of seven review articles and one comparative report of ILD registries in three European countries [9]. Two studies that identified racial differences in IPF epidemiology were excluded [10,11], as they did not represent overall incidence or prevalence calculations. The other disqualified original articles (n = 6) contained insufficient demographical data or insufficient cohort: article on prognostic factors and causes of death in Korean patients with IPF [12], Japanese case–control study of relationship between medical history and the risk of IPF [13], retrospective analysis of lower occurrence of IPF in Maori and Pacific Islanders [14], seasonal variation of mortality of pulmonary fibrosis [15], first report of the RIPID [16] and an Indian study of etiology of biopsy proven ILD [17].

**Figure 1:**
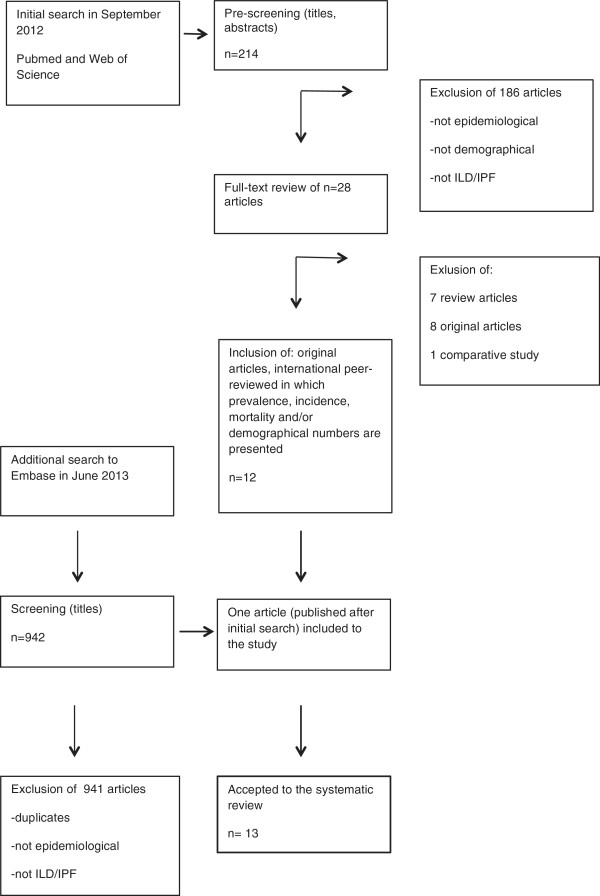
**Graphic presentation on article selection to this study.** Embase database became available to the authors after the initial search in 2012, therefore an additional study was performed in 2013.

An additional search was conducted in June 2013 to complement the systematic analysis. Embase was searched using the same queries as initially. 942 articles were found and titles were pre-screened by one author (JK). One additional article – published after September 2012 – was added to this systematic review. A summary of the selection process of articles is presented in Figure 1.

After the final evaluation, 13 articles were selected for data abstraction (Table 1.). Of the patient cohorts in these 13 articles, six were defined according to the ATS/ERS 2000 guidelines (Table 2).

**Table 1:** The articles included in this systematic review, categorized by the methodology used

**Type of study**	**Author**	**Year &journal**	**Study period**	**Cases (N)**	**Prev., Inc., mortality**
National registry data	Thomeer et al.	Acta Clin Belg 2001	1992-1996	72	Prevalence 1.25
					Incidence 0.22
	Tinelli et al.	Sarcoidosis Vasc Diffuse Lung Dis 2005	1998-2005	864	-
Questionnaires	Xaubert et al.	Sarcoidosis Vasc Diffuse Lung Dis 2004	10/2000-09/2001	197 (Inc)	Incidence 2.94
	Karakatsani et al.	Respir Med 2009	2004	189 (Prev)	Prevalence 3.38
				52(Inc)	Incidence 0.93
Analysis of pre-existing database	Hodgson et al.	Thorax 2002	1997-1998	1445	Prevalence 16-18
	von Plessen et al.	Respir Med 2003	1984-1998	158	Prevalence 19.7^1^-23.4^2^ (CFA)
					Incidence 4.3 (CFA)
					^1^31.12.1991, ^2^31.12.1998
	Gribbin et al.	Thorax 2006	1991-2003	920	Incidence 4.6
	Raghu et al.	Am J Respir Crit Care Med 2006	01/1996-12/2000	1943 (broad)	Prevalence, 42.7 (broad)
				387 (narrow)	Prevalence, 14.0 (narrow)
					Incidence, 16.3 (broad)
					Incidence, 6.8 (narrow)
	Olson et al.	Am J Respir Crit Care Med 2007	1992-2003	175,088	Mortality 50.8 (PF)
	Ohno et al.	Respirology 2008	2005	1322	Prevalence 3.44 (IIP)
	Fernández Pérez et al.	Chest 2010	1997-2005	47	Prevalence 27.9 (narrow)
					Prevalence 63 (broad)
					Incidence 8.8 (narrow)
					Incidence 17.4 (broad)
	Navaratnam et al.	Thorax 2011	mort.1968-2008	56,675	Mortality 2.54
			inc.2000-2008	2,074	Incidence 7.44
	Lai et al.	Respir Med 2012	1997-2007	418 (Prev)	Prevalence 0.7^1^-6.4^2^ (broad)
					Prevalence 0.5^1^-4.9^2^ (narrow)
					Incidence 0.6^1^-1.4^2^ (broad)
					Incidence 0.5^1^-1.2^2^ (narrow)
					^1^in 1998, ^2^in 2007

Prevalence (Prev) Incidence (Inc) and mortality/100,000.

**Table 2:** Characteristics of IPF patients’ demographics according to the studies included

**Study**	**Mean/median age at Dg**	**Male/Female**	**Total no of cases/**	**ATS/ERS 2000/2002**	**Before ATS/ERS 2000/2002**	**Re-evaluation**
			**% IPF of ILD**	**Dg criteria used**		
Thomeer et al. 2001	57 years	58/42%	362/20%		X Liebow 1975	
Tinelli et al. 2005	68 years	63/37%	3152/27%	X		
Xaubert et al. 2004			511/38.6%	X		
Karakatsani et al. 2009			967/20%	X		
Hodgson et al. 2002				X		X
von Plessen et al. 2003	69.2	45/55			X Turner-Warwick	X
Gribbin et al. 2006	71 years	62/38%			X	
Raghu et al. 2006					X	
Olson et al. 2007					X	
Ohno et al. 2008	65 years		1543/85.7%	X		
Fernández Pérez et al. 2010	74 years			X		X
Navaratnam et al. 2011	74 years	63/37%			X	
Lai et al. 2012					X	

% IPF of ILD. All of the ILD studies included sarcoidosis patients.

## Results

From the 13 articles chosen for this review, we identified three commonly-used methods for the evaluation of IPF epidemiology in a population, and used this knowledge to categorize epidemiological studies in order to simplify the comparison of heterogeneous population data (Table 1). First, we identified national registries of ILDs (here, two such studies were included [18,19]), where data collection was based on the activity of physicians. Second, two studies [20,21] were based on questionnaires for pulmonary physicians. A third, and by far the most common type of approach to IPF epidemiology, was the use of pre-existing databases, such as hospital databases and death registers, to calculate the overall prevalence and mortality of IPF [22-30]. Five studies included all ILDs (where IPF was usually the most common form) whereas in seven studies the focus was solely on IPF. In one report, the term pulmonary fibrosis, PF was used.

### National ILD registries

In Flanders—the northern part of Belgium—20 centers of respiratory medicine took part in the registration of ILD patients between the years 1992–1996 [18]. The twenty largest centers were relatively evenly distributed over the five provinces of Flanders. It was trusted that the registry consisted of a reliable percentage of the distribution of the ILD; it should be noted, however, that several centers did not participate in this collaborative study. Guidelines for diagnostic evaluation, therapy and follow-up recommendations of ILD were sent to pneumologists. Liebow’s classification from 1975 [31] was used for IPF definition. A total of 362 ILD cases were registered.

IPF was the second most common ILD (20%) after sarcoidosis. Of the 72 patients with IPF, 21 (29%) had biopsy-proven UIP, 10 patients had bronchiolitis obliterans organizing pneumonia (BOOP), nine had desquamative interstitial pneumonia (DIP), three had lymphocytic interstitial pneumonia (LIP) and in 29 cases (40%) the histology was not specified. The cases with biopsy proven IPF/UIP were identified according to the ATS/ERS 2000 guidelines [5]. Again male predominance was found, at 58.3% (with male/female ratio 1.4). The average age at diagnosis was 57 years. In Flanders, the registry data suggests an incidence of 0.22/100,000 and prevalence of 1.3/100,000 of IPF, which is significantly less than in comparable studies [31] (Table 1 and 2). It was speculated by the authors that the low prevalence of IPF was primarily due to a selection bias by the type of registry or applied diagnostic procedures.

The Italian register for diffuse infiltrative lung disorders (RIPID) was established in 1998 [19]. Registration was voluntary, and implemented by 138 physicians in 79 centers located in all 20 regions of Italy. The greatest numbers of these cases were from northern Italy (74.3%), and a minor fraction from central Italy (11.3%) and southern Italy (14.4%). The accuracy of the data in regard to the international diagnostic criteria (ATS/ERS 2000) could not be verified as it relied on the quality of the input received from the different physicians in different institutions. Up to January 18, 2005, a total of 3,152 patients had been included. IPF was the second most common ILD after sarcoidosis consisting of 27.4% of patients (n = 864). Until 1999–2000 the number of IPF cases diagnosed was rising in Italy, but during 2000–2005 this remained constant. There was a slight male predominance in this data (62.6%), which is a usual finding in all studies on IPF [32]. The mean age of onset was 68 years. Epidemiological parameters, such as incidence and prevalence, were not accurately calculated since the size of the population (of the participating centers) was not available.

Both of these national registry studies rely considerably on the activity of physicians in reporting patient numbers and data, and studies performed in this way should always be interpreted knowing of this bias, that results in underestimation of patient numbers. Registry cohorts like these, are, however important in the determination of patient clinical course and the possible differences in the management of patients in different centers.

### Questionnaire-based surveys of ILD in Greece and Spain

Two methodologically similar articles were found from Greece and Spain, where a standardized questionnaire was sent to respiratory medicine centers in order to obtain information on patients’ clinical data, procedures used to establish the diagnosis and the exact diagnosis.

In Spain, a registry of incident cases of ILD was set up from October 2000 to September 2001 [20]. A total of 37 centers were contacted, but only 23 (62.1%) replied and participated. These 23 centers covered a population of 6.7 × 10 ^6^ inhabitants. A standardized questionnaire was sent to the centers. An ATS/ERS consensus statement from 2002 was applied for classifying ILDs. A total of 511 cases of ILD were registered. Idiopathic pulmonary fibrosis was the most frequent disease, covering 197 patients/38.6% of all ILD patients; thus, according to our calculation, this results in an incidence of 2.94/100,000. The diagnosis was established by a surgical lung biopsy in 31.5%, and clinical criteria including HRCT scanning in 68.5%.

In Greece, departments of Pneumonology were contacted, and pneumonologists were asked to complete a questionnaire for all ILD patients who were alive in 2004 (prevalent case), as well as for every new incident case diagnosed during 2004 [21]. Centers covering about 60% of the Greek adult population (5.6 million inhabitants) replied and participated in the study. For the classification of the IIPs the ATS/ERS consensus from 2002 was used. Due to funding limitations, no reevaluation of the cases could be done. A total of 967 cases of ILD were reported during 2004. Idiopathic pulmonary fibrosis accounted for about 20% of all ILDs. Open lung biopsy was performed in 18.5%, and a VATS biopsy in 13.2%, of IPF cases. The number of prevalent cases (IPF/UIP) was 189 (19.5%), and the number of incident cases was 52 (20.1%) The prevalence of IPF in 2004 in Greece was calculated to be 3.38/100,000 and incidence 0.93/100,000.

Due to the low response rates and lack of monitoring of the centers that have attended the study, data derived from these two studies should be interpreted with caution.

### Analysis of health care system registries

In Finland, the prevalence of sporadic and familial IPF was evaluated during the years 1997–1998 [22]. All Finnish pulmonary clinics (N = 29) were contacted, and hospital databases were screened for ICD-10 diagnosis J84.1. In selected hospitals (N = 13) the diagnoses were reevaluated by a specialist in pulmonary medicine using the ATS/ERS 2000 recommendations. The screening of hospital diagnosis registries showed to be sensitive but highly non-specific, since 23-51% of all cases were excluded. All five Finnish university hospitals and eight smaller district hospitals were chosen for the study, to ensure that all geographical areas were covered. The total number of patients with IPF was extrapolated using the screened and re-evaluated patient data. The study group identified 1,445 inpatients or outpatients with the diagnosis of IPF or fibrotic alveolitis. In 90% of the patients, lung involvement was assessed by HRCT scanning and 28% of IPF patients had histological verification. No predominance of either sex was observed. The prevalence of IPF in Finland was estimated to be 16–18/100,000. Because the majority of the IPF cases were not histologically verified, it was discussed by the study group that this could be a source of uncertainty in the prevalence estimates. The strong point in this particular study was the reliability of population data due to a comprehensive up-to-date national population registry, and a high-standard health care system that is available to all citizens. The weaknesses of this study were, that it was performed very soon after the diagnostic criteria were changed, and the cohorts did not include the whole population.

In Norway, the incidence and prevalence of physician-diagnosed and hospitalised cryptogenic fibrosing alveolitis (CFA) was studied during 1984–1998 [23]. Thus, the study started long before HRCT was available in all hospitals and before ATS/ERS 2000 obtained. The diagnostic clinical criteria by Turner-Warwick [33] was used. The study population (Bergen hospital district) covered total of 250,000 inhabitants. A computer-aided search of the hospital registers was carried out. Two physicians abstracted data and the following cases were excluded: pulmonary fibrosis associated with collagen vascular disease, intake of cytotoxic and other potentially fibrogenic drugs, radiotherapy and exposure to asbestos as well as silica, sarcoidosis and allergic alveolitis. The search resulted 376 patients of which 158 were defined as incident CFA cases. Mean age at diagnosis of incident cases was 69.2 years and 45% were men. Norwegian women are among the heaviest smokers in the world. Smoking - as a probable risk factor for CFA - may explain the female predominance, especially because women are at higher risk developing respiratory disease. The average annual incidence hospitalised CFA was 4.3/100 000 inhabitants. The prevalence was 23.4/100 000 and 19.7/100 000 by 31.12.1998 and 31.12.1991 respectively. Median survival of incident cases was 4.1 years. It should be kept in mind that due to the diagnostic criteria used the whole spectrum of idiopathic pulmonary fibrotic disorders was included including i.e. nonspecific interstitial pneumonia (NSIP). This study represents a very small, but carefully and reliably analysed patient cohort, but due to the relatively small patient number from the same area and genetic background, the epidemiological parameters should be interpreted with caution. However, compared with the similarly performed study of Hodgson et al. [22], the prevalence of IPF is at a comparable level.

In the United Kingdom, a computerized longitudinal general practice database (The Health improvement Network, THIN) was analyzed to estimate the incidence of IPF and sarcoidosis between 1991–2003 [24]. The term IPF was used to describe clinical diagnosis because only a small majority of patients were diagnosed with an open lung biopsy. IPF patients were identified with diagnostic terms: “cryptogenic fibrosing alveolitis” and “idiopathic fibrosing alveolitis”. Patients were included if they had at least one recorded IPF diagnosis, their first IPF diagnosis was recorded at least 12 months after the patient’s first mark in the database and they were aged at least 40 years when they received the first diagnosis. The IPF patient cohort was analyzed with a matched general-population control cohort. There were 920 patients with IPF (≥ 40 years at diagnosis) and 3,593 matched controls were identified. The mean age at diagnosis was 71 years, and 62% were male. The overall incidence rate for IPF was 4.6/100,000. The crude rates also increased progressively over time and tended to be higher in the northern and western regions of the UK. After controlling for the effects of sex, age and geographical region, the estimated annual increase in the incidence of IPF was 11%. The 3- and 5-year survival percentages for patients with IPF were 57% and 43%, respectively and the equivalent percentages in the comparison cohort were 88% and 81%. The authors questioned the validity of the IPF diagnoses, and despite the study design some of the incidental IPF cases could be prevalent. The main strength of this study was the large size of the patient cohorts, and the coverage of the cohort to the whole population. Compared to other studies, however, the prevalence is lower, suggesting that not all IPF patients were found using this particular method. Patient data were not re-evaluated, which can be considered as a major weakness in this study.

In the United States, the annual incidence and prevalence of IPF was estimated by utilizing a large health care claims database from 01/1996–12/2000 [25]. IPF patients were recognized through both broad and narrow definitions. The broad case definition criteria included age, ≥ 18 years, one or more medical claim with a diagnosis code for IPF (ICD-9-CM 516.3, not specific for IPF), no medical claims with a diagnostic code for any other ILDs on or after the date of the last medical claim with a diagnosis code for IPF. The narrow case definition included the broad case definition and one or more medical claims with procedure codes for a surgical lung biopsy, a transbronchial lung biopsy, or a computed tomography of the thorax, on or before the date of the last medical claim with a diagnosis code for IPF. From 2.2 million men and women, 1,943 patients met the broad case definition; among which, 387 met the narrow case definition. Based on the broad case definition, the prevalence of IPF was estimated to range from 4.0/100,000 (age, 18–43 years) to 227.2/100,000 (≥75 years). Incidence ranged from 1.2 to 76.8/100,000, respectively. Based on the narrow definition, prevalence was estimated to range from 0.8/100,000 (age18–34 years) to 64.7/100,000 (≥75 years) and incidence from 0.4 to 27.1/100,000 respectively. Extrapolating these rates to the overall population of the United States, prevalence was estimated to be 42.7/100,000 and incidence 16.3/100,000 using the broad criteria. With the narrow criteria, prevalence was estimated to be 14.0/100,000 and incidence 6.8/100,000. Both prevalence and incidence was generally higher among men than women. According to the authors, the limitations of this study were that the database included only health-plan members in selected geographic regions of the United States, data were collected for the most part before the ATS/ERS consensus and statement of the classification in 2002. The study population consisted solely of persons that had insurance, and who thus may differ systematically from the rest of the population in terms of their health status and/or medical-care utilization and expenditures. When this study is compared with other studies presented in this review, the narrow criteria for IPF result in very similar prevalence rates as the health care system studies ie. in Finland and Norway [22,23]. Due to a different kind of health care system organization (publicly versus privately funded) these studies are not comparable.

In another study conducted in the United States, mortality to pulmonary fibrosis (PF) was studied from 1992–2003 [26]. The National Center for Health Statistics’ (NCHS) databases was screened to calculate mortality rates and examine the underlying cause of death. From 1992 to 1998 NCHS coded conditions related to death with ICD-9, and began using ICD-10 from 1999. From 1992 to 2003, there were 28,176,224 deaths in the United States. Of these deaths, a total of 175,088 records contained a diagnostic code for pulmonary fibrosis. Using the entire subset of 175,088 decedents, the age-adjusted mortality rate increased to 29.4% in men and 38.1% in women during the study period. When connective tissue disease, asbestosis and radiation fibrosis were excluded from the original analysis, the age-adjusted mortality rate increased over the study period to 28.4% in men and 41.3% in women. In the third analysis, the most rigorous definition in PF was used, and records that contained codes for both PF and any other condition known to cause PF were excluded. Analysis now showed mortality rates increasing to 27.5% in men and 40.8% in women. The authors speculated that persistently rising mortality rates might reflect an increase in clinical recognition of pulmonary fibrosis during the study period. The average age- and sex-adjusted mortality rate to PF was 50.8/100,000. Mortality rates varied greatly by state: states with the lowest mortality were New Jersey, New York, Nevada and the District of Columbia. The highest mortality rates were found from New Mexico, Vermont, North Carolina and South Carolina. The authors speculated that this geographic variation might have developed due to real differences between races, or there might be differences in the identification of the disease, or differences in the rate of the use of diagnostic tests. In addition, environmental factors were proposed to have an effect on the pathogenesis of the disease. The most common cause of death in this patient group was PF itself. The obvious limitations in this study are that it relies on accuracy of a particular death registry and that the patients included are not reevaluated according to the modern diagnostic criteria. The important value of this study is, however the increases in mortality, which should be assessed in future studies.

Clinical records were also utilized in Japan to gain epidemiological data of IIP [27]. All IIP patients who delivered medical benefits during 2005 were included in the study. As medical benefits are examined in each prefecture, clinical records were collected and submitted to the Disease Strategy Section, Health Bureau, Ministry of Health, Labor and Welfare and then stored in a database in the Ministry. It is noteworthy that IIP conditions, for which medical benefits were delivered, corresponded to severe cases; milder cases of IIP were thus not included to this study. In 2005, medical benefits were delivered to 4,396 patients with IIP. Of these patients, clinical personal records were available for 1,543 patients. A total of 1,322 patients (85.7%) had IPF of which 545 were newly diagnosed patients and 777 updated patients. The diagnosis was confirmed by surgical lung biopsy in 12% of the patients. The mean age at disease onset was 64.5 years. There was no gender difference. The prevalence of IIP was estimated to be 3.44/100,000. According to this, the prevalence of IPF would then be 2.95, which is likely to be an underestimation, as mild disease forms were not recorded. Again, this study shows that cultural differences in health care registries may result in very dissimilar results when epidemiological data were collected.

In Olmsted County, Minnesota, during a nine-year period (01/1997–12/2005) residents were screened, and those with possible IPF were identified [28]. These cases were identified through the diagnostic index (e.g. ICD-9-code) using the resources of the Rochester Epidemiology Project (REP), which links together medical diagnosis and procedure information across all medical providers in the county. Medical care is essentially self-contained within Olmsted County. Using REP ensured that 100% of the cases within a defined geographic region were approached. IPF cases were identified by two methods: 1) evidence of UIP on surgical lung biopsy specimens or definite UIP pattern on HRCT (narrow-case finding criteria) and 2) evidence of UIP on surgical lung biopsy specimens or a definite or possible UIP pattern on HRCT (broad-case finding criteria). The first method met all major and minor ATS/ERS 2000 criteria in the absence of a surgical lung biopsy, and the second method included all patients in the first method and a subgroup that met ATS/ERS criteria but in whom the HRCT features were characterized as possible for UIP. For each patient identified, their complete medical record was reviewed by a trained data abstractor and one of the investigators. In addition, for each case identified the HRCT images were reviewed by two thoracic radiologists and ranked as definite or possibly UIP or being unlikely UIP. All lung biopsy specimens were re-analyzed by a pulmonary pathologist to confirm the diagnosis of UIP. For 596 patients screened, 47 new cases of IPF were identified which could be viewed as too small for reliability. Of these 47 patients, 24 met the defined narrow criteria. The mean age at diagnosis was 73.5 years, and 29% of patients had histologically proven UIP. The age- and sex-adjusted incidence was 8.8/100,000 (narrow criteria) and 17.4/100,000 (broad criteria). The incidence rate was higher in men than in women, and highest among those aged 70–79 years. The adjusted incidence rate of IPF decreased during the study period. The prevalence of IPF in Olmsted County (on December 31, 2005) was 27.9/100,000 (narrow criteria) and 63/100,000 (broad criteria). During the 9-year observation period, the median survival for narrow and broad criteria was 3.47 years and 4.37 years, respectively. Although this report presented a very small cohort of patients, it was one of the studies in which reevaluation was conducted in a very reliable manner, and thus this study might represent the most reliable estimates of IPF epidemiological parameters.

In the United Kingdom, death registers and general practice databases were used to investigate mortality (1968–2008) and incidence (2000–2008) of IPF [29]. The diagnostic classifications used in both datasets were not made according to the ATS/ERS 2000 classification, which may result in the overestimation of cases. There were 56,675 deaths attributed to IPF clinical syndrome (IPF-CS) in England and Wales from 1968 to 2008. Three different ICD codes were used to code death from IPF-CS, namely ICD-8 code 517, ICD-9 codes 516.3, 515 and ICD-10 code J84.1. The overall mortality rate standardized to the 2008 UK population was 2.54/100,000. Mortality rates increased significantly during the study period —from 0.92/100,000 during 1968–1972 to 5.10/100,000 during 2006–2008 being higher in men and the older age groups. In the same study the sub analysis of the ICD-9 codes showed similar trends. The primary care database recorded by UK general practitioners—The Health Improvement Network (THIN)**—**was also screened for diagnoses of IPF-CS between 2000–2008. Cases with a co-existing connective tissue disease, and individuals who had co-existing diagnoses of extrinsic allergic alveolitis, asbestosis, pneumoconiosis and sarcoidosis were excluded. The search resulted in a total number of 2,074 incident cases which equates to an incidence of 7.44/100,000. The majority (63%) of new cases were men. The mean age at diagnosis was 74.3 years. After adjusting for age, sex and health authority, the estimated annual increase in the incidence of IPF-CS was 5%. The overall median survival for this cohort was 3.03 years. The advantage in the methodology used here is the combination data derived from of two different and independent registries, but the results are handicapped with the lack of reevaluation of the diagnostic accuracy of these registries.

In Taiwan, a large population-based study was conducted to collect epidemiological information of IPF among Asians [30]. The data were collected from two Taiwanese governmental organizations –the Taiwan National Health Insurance (NHI) system and the national mandatory death registry system – during 1997–2007. The NHI covers and protects almost all Taiwanese, and has detailed information on health services (almost all outpatient visits and hospital administrations), procedures and prescriptions, their payments and times of use. Patients’ diagnoses were coded using the ICD-9. For comparative purposes, the same definitions of IPF were used as in the study of Raghu et al. [25]. The broad definition consisted of the following three conditions: 1) there was at least one NHI claim with IPF diagnosis (ICD-9-CM code 516.3) between January 1, 1997 and December 31, 2007, 2) for at least one NHI claim with the diagnostis of IPF, there were no NHI claims for any other ILDs after the day of IPF diagnosis, 3) The patient’s age was ≥ 18 at the time of confirming IPF. The conditions for the narrow definitions were: 1) all conditions for the broad definitions were met, 2) for at least one NHI claim with IPF diagnosis, there was at least one NHI procedure for surgical lung biopsy, or computed tomography of the thorax before or on the day IPF diagnosis.

The estimated prevalence, based on the broad case definition, ranged from 0.7 in 1998 to 6.4 in 2007. According to the narrow case definition the estimated prevalence was 0.5 in 1998 and 4.9 in 2007. The annual incidence was calculated to be 0.6 in 1998 to 1.4 in 2007 (broad definition,). The incidence ranged 0.5 in 1998 to 1.2 in 2007 (narrow case definition, Table 1). Both prevalence and incidence increased during the study period irrespective of the case definition. Both prevalence and incidence were generally higher among aged people (over 75 years) and men. The median survival time after IPF diagnosis was 0.9 years or 0.7 year for the broad and narrow definitions, respectively. The authors pointed out, that incidence and prevalence were found to be relatively lower in Taiwan than in western countries. This was suspected to be due to differences in diagnostics or in ethnics. Survival of the IPF patients was much shorter than in the United States [28]. It was speculated that this could be due to a delay in diagnosis; most of the Taiwanese patients were diagnosed at advanced disease stages. The major advantage in this study is the use of a health care registry that covers the whole population. Unfortunately, the identified patients were not reevaluated, which affects study reliability and prevents comparisons to other similar studies.

## Discussion

Our systematic search on IPF epidemiological parameters resulted in 13 eligible original articles published after 2001, of which six used the ATS/ERS 2000 diagnostic criteria or classification recommendations of IIP from 2002. Our results indicate that there exist only a few reports on IPF epidemiology according to the modern diagnostic criteria, and that all studies had either major methodological limitations or a small cohort of patients, which makes the comparison of epidemiological parameters in these studies very difficult or impossible. No original articles have been published so far on IPF prevalence according to the new diagnostic criteria (ATS 2011) or from IPF registries [8,34]. The use of uniform, international diagnostic guidelines is of utmost importance in the field of IPF medicine, and will most likely enable the collection of more accurate and comparable data in the future. It is, however, surprising that quite a few articles have been published after the publication of the first diagnostic criteria in 2000, yet they did not make use of these criteria by means of the reevaluation of patient data. Data reevaluation requires access to a large set of patient data, including histological lung biopsies and original CT scans, and often requires a complete search of all medical records, perhaps making it too laborious and costly. However, as our literature search showed, this has been done in restricted patient cohorts [22,23,28], and could well be standardized as a method for future studies. Uniform electronic patient journals and digital image archives may offer some help in this setting, making the collection and evaluation of epidemiological data easier for scientists in the future. Thus it is important to collect data in such a way that even if the diagnostic criteria change, the data could still be used.

In addition to the identification of epidemiological parameters, our primary aim was to identify plausible and reliable methods in studies related to IPF epidemiology, as the field of IPF medicine is undergoing considerable change due to specific drugs that are entering clinical use, and many nationwide registry databases which are currently being built. Various and very heterogeneous methods are being used by IPF researchers, and so far the data from these studies is not comparable between countries due to this variation. We identified three categories or strategies for collecting IPF epidemiological data: nationwide registries for IPF or ILD, such as the Italian registry; questionnaires for health-care professionals; and systematic searches from existing databases. From these methods, questionnaires seemed to produce the lowest prevalence rates compared to national registries and/or searches from pre-existing databases. The coverage of pre-existing databases might be high in cases such as those shown in the study of Navaratnam [29], but the downside of this kind of study rationale is the high number of patients that have been misdiagnosed i.e. having a high number of “false positive” cases.

The number of incidental and prevalent cases varied greatly in the presented studies (prevalence from 0.5 [30] to 27,9/100,000 [28]). In some of the selected articles e.g. [18,21], it was speculated that this variation may be due to real differences between countries. Our results suggest that, primarily, the differences in epidemiological parameters are more likely to be a result of the heterogeneous methods used than true geographical differences in IPF epidemiology. The fact that studies which used very similar methodologies resulted in similar results (for example the questionnaire studies, where the incidence ranged from 0.93 to 2.94/100,000) supports this view. However, even these studies are not comparable as the questionnaires used, and the strategies for obtaining answers, were different. The possible differences in results between epidemiological studies are summarized in Table 3. One shared problem in all methods is the difficulty of finding the right denominator to epidemiological calculations. For example in the registry study of Thomeer et al. [18] low prevalence and incidence rates may be due to overly high denominator; not all but 20 centers participated to the study which doubtfully covers the whole Flanders population. In addition, the lack of a specific diagnostic coding for IPF, that would differentiate it from other ILDs has not existed before the 2013 version of the ICD-10 diagnostic system.

**Table 3:** The possible reasons for the high variability of epidemiological parameters observed in the different studies

**Epidemiological parameter**	**Reason for variability**
Prevalence	Definition of disease
	Diagnostic guidelines
	Healthcare system related
	Methodological differences
Incidence	Improved diagnostics
	Improved health care system
	Ageing population
	Drug availability
Mortality	Clinical recognition
	Changes in diagnostic coding
M/F ratio	Occupational exposures
	Population smoking habits

Changes in diagnostic criteria may also explain some of the observed variation. Studies implemented before the ATS/ERS 2000 guidelines [5] have shown the lowest prevalence at 0.5 in Taiwan [30] and the highest in Norway at 23.4 [23]. After the publication of more definitive diagnostic criteria, prevalence has varied between 3.38 [21] to 27.9 [28]. Until the 1990s, the Liebow classification of idiopathic interstitial pneumonia (IIP) was used, which did not include NSIP [35]. NSIP was described in 1994 [36], and subsequently a new proposal of the classification of IIPs, which also included NSIP, was presented by Katzenstein and Myers in 1998 [37]. Until this proposal, NSIP was probably diagnosed mostly as IPF or, in some cases, also as desquamative interstitial pneumonia (DIP). The international guidelines of IPF and IIP in 2000 and 2002 recommended that NSIP should be separated from IPF, which has been gradually implemented into the clinical practice during the 2000s. Thus, it is likely that most of the studies in the present review may have also included patients with NSIP in addition to IPF, at least in the beginning of the study periods, because of the historical background and the change in classifications. Currently a new classification of IIPs is being updated which will include a new category, namely unclassifiable IIP [38], which has been shown to comprise about 10% of an ILD cohort [39]. It may be reasonable to assume that in the above-mentioned epidemiological studies not only the patients with NSIP, but also those with unclassifiable fibrosis, may have been included.

The international disease classifications have also reflected the lack of a clear, systematic classification of ILD:s: only in the latest version of ICD-10 from 2013, there has been a separarate subclassification for IPF. It is to be expected, that studies performed after this disease classification has been taken into use, and in countries where the population registries are most reliable, will yield high-quality data on IPF epidemiology. There are already preliminary reports on several national registry studies, which hopefully will yield comparable data on IPF epidemiology in different geographical and cultural areas [7,40,41].

## Conclusions

Overall, it can be concluded that the existing databases present an overestimation of patient numbers, whereas registries and questionnaire-type studies result in an underestimation of patient numbers. The acquirement of accurate epidemiological data is crucial in elucidating the natural course and prognosis of IPF in different populations, and can lead to a better understanding of the disease etiology. However, if epidemiological data are collected, it should include a broad systematic screening of registries in the health care system, and a specific reevaluation of the patient records, especially the evaluations of pulmonary pathologists and radiologists. Novel electronic databases will hopefully offer more comparable data in this setting in the near future.

## Abbreviations

BOOP: Bronchiolitis obliterans organizing pneumonia; DIP: Desquamative interstitial pneumonia; HRCT: High resolution computed tomography; IIP: Idiopathic interstitial pneumonias; ILD: Interstitial lung diseases; IPF: Idiopathic pulmonary fibrosis; LIP: Lymphocytic interstitial pneumonia; NSIP: Nonspecific interstitial pneumonia; PF: Pulmonary fibrosis; UIP: Usual interstitial pneumonia; VATS: Video-assisted thoracic surgery.

## Competing interests

None of the authors have competing interests related to this work.

## Authors’ contributions

JK: reviewed the original articles included in this work, collected data from these articles, and drafted and wrote the manuscript; E-RS: reviewed the original articles included in this work, and wrote the manuscript, UH:^,^ revised the manuscript, RK: presented the original idea for this work, reviewed the original articles included in this work and participated in writing the manuscript, MM; reviewed the original articles included in this work, and contributed in the writing of the manuscript. All authors read and approved the final manuscript.

## Pre-publication history

The pre-publication history for this paper can be accessed here:

http://www.biomedcentral.com/1471-2466/13/53/prepub
